# Symptom Cluster Research in Women with Breast Cancer: A Comparison of Three Subgrouping Techniques^*#^

**DOI:** 10.4236/abcr.2013.24018

**Published:** 2013-10

**Authors:** Angela R. Starkweather, Debra E. Lyon, R. K. Elswick, Alison Montpetit, Yvette Conley, Nancy L. McCain

**Affiliations:** 1 School of Nursing, Virginia Commonwealth University, Richmond, USA; 2 School of Nursing, University of Pittsburgh, Pittsburgh, USA

**Keywords:** Breast Neoplasms, Cluster Analysis, Symptom Clusters, Psychoneurological Symptoms

## Abstract

**Aims:**

To examine how symptom cluster subgroups defined by extreme discordant composite scores, cut-off scores, or a median split influence statistical associations with peripheral cytokine levels in women with breast cancer.

**Background:**

Systemic cytokine dysregulation has been posited as a potential biological mechanism underlying symptom clusters in women with breast cancer. Symptom characteristics may play an important role in identifying cytokines of significant etiological importance, however, there is no consensus regarding the ideal subgrouping technique to use.

**Design:**

A secondary analysis of data collected from a cross-sectional descriptive study of women with stage I-II breast cancer was used to examine and compare the relationships between peripheral cytokine levels and symptom subgroups defined by extreme discordant composite scores, cut-off scores, or a median split.

**Methods:**

Participant symptom scores were transformed into a composite score to account for variability in symptom intensity, frequency and interference. Cytokine levels in subgroups defined by composite scores within the highest and lowest 20% were contrasted with those composed from cut-off scores and a median split.

**Results:**

Subgroups defined by the composite score or cut-off scores resulted in similar statistical relationships with cytokine levels in contrast to the median split technique. The use of a median split for evaluating relationships between symptoms clusters and cytokine levels may increase the risk of a type I error.

**Conclusion:**

Composite and cut-off scores represent best techniques for defining symptom cluster subgroups in women with breast cancer. Using a consistent approach to defining symptom clusters across studies may assist in identifying relevant biological mechanisms.

## 1. Introduction

More than 220,000 women will receive a diagnosis of breast cancer (BC) this year in the United States [1]. While advances in treatment have dramatically improved the rate of survival for women with BC, a large proportion of women undergoing treatment report multiple co-occurring symptoms that can be a significant source of distress [2,3]. Research has shown that these multiple co-occurring symptoms, or symptom clusters, can have a profound negative impact on quality of life [4,5]. Specifically, symptoms of fatigue, depression, sleep disturbances, and pain are prevalent across stages of disease and BC treatment [6]. Women who report more symptoms at diagnosis have a greater symptom burden during chemotherapy [7-9] and significantly lower quality of life [10,11]. Besides the number of symptoms reported, symptom severity has also shown an inverse relationship with quality of life [12].

Although there is ample evidence showing a high prevalence of symptoms clusters among women with BC and negative effects on quality of life, there have been few studies focused on exploring potential biological mechanisms associated with these symptoms. Identification of the biological mechanisms underlying the development and persistence of this symptom group, known as the psychoneurological (PN) symptom cluster, could lead to more targeted symptom management strategies [13].

A putative mechanism of PN symptoms in women with BC involves ongoing inflammation resulting from dysregulation of the hypothalamic-pituitary adrenal (HPA) axis. Support for the connection between HPA axis dysfunction, inflammation and symptom clusters is derived from the research describing cytokine-induced sickness behavior in animal models and human studies [14]. The sickness behavior model is relevant to symptom research in BC because the disease process and treatments, including chemotherapy and radiation, are associated with cytokine elevations [15,16].

Although there are some evidences that supports a relationship between PN symptoms and alterations in neuroendocrine hormones and proinflammatory cytokines in women with BC, the findings have not been consistent [17,18]. This may be due to differences in how the symptom cluster is measured across studies. Symptom clusters are often selected a priori and quantified based on the presence or absence of each symptom which can mask wide variations of symptom frequency, severity and duration within the symptom cluster group. Small sample sizes and heterogeneous samples with different stages of BC or treatment may further decrease statistical power to detect significant relationships among symptoms and biological factors.

In contrast, homogeneity of the symptom cluster subgroup can be increased by using symptom domains (prevalence, severity, frequency, interference) to identify subgroups with similar symptom characteristics. For instance, among 191 individuals with cancer receiving treatment, PN symptom clusters based on symptom severity revealed four subgroups—all low symptoms, high pain low fatigue, high fatigue low pain, all high symptoms [6]. This method allows for the identification of a predominant symptom of the symptom cluster, in this case, fatigue, pain, or all symptoms. Another approach using cut-off scores for each symptom has been described based on symptom prevalence and severity [11].

Providing a basis to distinguish those who definitely have the symptom cluster from those who definitely do not, an extreme discordant analytic approach can be used to increase statistical power to detect relevant biological mechanisms. This is similar to the sampling designs used by researchers in genetic epidemiology that are used to discover causal variants [19]. By selecting extreme discordant subgroups of the symptom cluster (e.g., all high vs. all low symptoms or high vs. low predominant symptom) it would be expected that biological factors of etiological importance to the symptom(s) would be significantly different between groups. In order to examine how the various subgrouping techniques influence the statistical associations with cytokine levels a secondary analysis was performed on an existing dataset of symptom profiles and peripheral cytokine measurements in women with BC.

## 2. Methods

A secondary analysis of 128 women with BCA was subsequently undertaken to contrast the application of extreme discordant subgroups defined by composite scores, cut-off scores or a median-split. The dataset was part of a larger research study carried out in two university health systems in the mid-Atlantic region from 2004-2009. Women diagnosed with Stages I-II BCA were approached about study participation at 4 weeks after lumpectomy or mastectomy, before receiving their first dose of chemotherapy. This time point was selected because it provided an adequate duration of time after surgery for return of baseline immune parameters.

Inclusion criteria were women over the age of 18 years diagnosed with Stages I-II BCA after lumpectomy or mastectomy and fluency in English. Exclusion criteria included a past medical history of cancer or immune-related disease (*i.e.* multiple sclerosis, HIV, lupus), use of anxiolytics or anti-depressants, or regular use of anti-inflammatory medications. All participants verbalized understanding and gave informed consent to the research protocol, which was approved by the university's institutional review board.

### Procedures

At the time of consent, participants completed demographic forms and self-report questionnaires. After completing the questionnaires, a blood sample was collected from each participant using a standard phlebotomy protocol into a serum separator vacutainer without anticoagulant and the vial was transported on ice directly to the laboratory for processing. Sera were separated by centrifugation, and all specimens were aliquoted immediately, frozen, and stored in a −70°C freezer until batch processing.

### Symptom measures

Fatigue and sleep disturbance were measured on the Symptom Experience Survey, with 3 items indicating the frequency, severity and distress of each symptom using a Likert scale from none (0) to severe (4) [20]. A total score ranging from 0 - 12 was used for the analysis. Depressive symptoms were measured by the Center for Epidemiological Studies Depression Scale (CES-D) [21] and the Brief Pain Inventory-Short Form [22] was used to measure pain severity and interference. These measures have well-established reliability and validity for adult patients with no cognitive impairment in studies of cancer and its symptoms [23,24].

### Cytokine levels

Plasma concentrations of cytokines were measured with the Bio-Plex Human 17-Plex (Bio-Rad, Hercules, CA). This standardized kit includes coupled beads, detection antibodies, and standards for the detection of IL-1β, IL-2, IL-4, IL-5, IL-6, IL-7, IL-8, IL-10, IL-12, IL-13, IL-17, G-CSF, granulocyte-macrophage colony-stimulating factor, interferon-gamma (IFN-γ), monocyte chemoattractant protein-1, macrophage inflammatory protein-1β, and TNF-α. After incubation, contents of each microplate well were drawn into the Bio-Plex array reader, and precision fluidics align the beads in a single file through a flow cell, where two lasers excite the beads individually. High-speed digital signal processors and Bio-Plex Manager software (Bio-Rad; Hercules, CA) record the fluorescent signals simultaneously for each bead. Levels of CRP were determined using a high-sensitivity enzyme-linked immunosorbent assay (ELISA) assay (ALPCO Diagnostics, Salem, NH). Sensitivity of all measurements = 10 pg/mL.

### Subgrouping techniques

Cut-off scores for each symptom assessment measure were derived from the literature and were correlated with moderate to severe symptom intensity, frequency, and interference. A cut-off score of 6 or above was applied to indicate moderate fatigue, measured on the Symptom Experience Survey. Depressive symptoms were cut-off score at ≥16, the traditional indicator for probable depression on the CES-D. A cut-off score of 6 or above was applied to indicate moderate sleep disturbance, measured on the Symptom Experience Survey. A cut-off score of ≥4 was used for the Brief Pain Inventory-Short Form, indicating moderate pain severity and interference. However, there were only three participant scores that met the cut-off scores applied to pain and sleep disturbances. Because the sample scores for pain and sleep disturbances were generally low, the analysis proceeded with the symptom cluster of fatigue and depressive symptoms. Participants with both symptom scores above the cut-off were designated as the high-symptom cluster group while participants with both symptom scores below the cut-off were designated as the low-symptom cluster group.

To standardize symptom scores among the instruments used, the symptom scores were transformed to a 1 - 100 scale. This transformation provided equally weighted scales so that each score value contributed equally to the sum score. A composite symptom cluster score was computed for each individual by summing the two transformed scores. The subgroup of women with the highest composite symptom cluster score, defined as the top 20%, were chosen for the extreme discordant subgroup analysis. In the same manner, the 20% of women with the lowest composite symptom score comprised the low symptom cluster group. With a sample of 128 women with BCA, the top 20% (*n* = 25) with the highest composite symptom cluster score were selected. Of these, 20/25 (80%) met the cut-off scores for fatigue and depression, 25/25 (100%) met the cut-off for depression, and 20/25 (80%) met the cut-off score for fatigue. In contrast, when using the cut-off scores for fatigue and depression as inclusion in the extreme symptom cluster phenotype, there were 24 participants who fell within this group (blue line).

**Figure 1:**
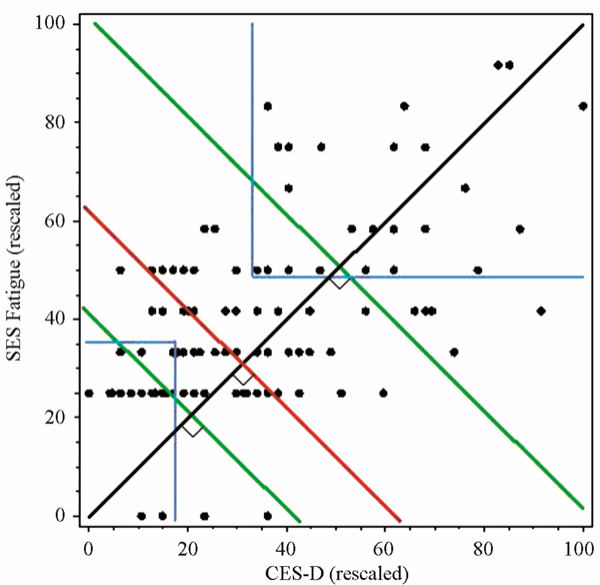
Comparison of subgroups defined by composite scores, cut-off scores, or median-split. Clusters defined by 20% of the highest and lowest composite scores = green lines; Clusters defined by both above cut-off scores = blue lines; Clusters defined by median split = red line.

A visual representation of the subgrouping technique is provided in Figure 1. Subgroups were determined by the level of dependence between the two symptoms. If the symptoms were highly dependent, the cumulative symptom score fell above the upper 20% line (second green line), whereas when the symptoms were independent, the cumulative symptom score was below the lower 20% line (first green line). Scores that were not highly dependent or independent were in between the two green lines.

Thus, while the composite symptom cluster score method included some participants who had symptom scores below the cut-off range, the cut-off score method included some participants who did not reach a composite symptom score within the highest 20% of the sample. Using a composite or cut-off score to identify a predominant symptom (fatigue or depression) or cluster of symptoms (fatigue and depression) results in extreme discordant subgroups, which can maximize statistical power. Based on this model, cytokine profiles were examined and compared using the composite symptom cluster score, symptom cut-off scores, and a median split of the sample.

## 3. Results

This sample of 128 women had a mean age of 47.7 years (*SD* 7.7, Range 27 - 63), was mainly Caucasian (62.5%) and was slightly more post-menopausal (53%) than pre-menopausal (46.9%). The majority of women underwent mastectomy (65.6%) as opposed to lumpectomy (34.4%).

Findings demonstrated similar statistical relationships between the symptom cluster and cytokine levels when using the composite symptom cluster score or cut-off scores (Tables 1 and 2). A significant difference between the high and low composite symptom score subgroups was found for interleukin (IL)-6 and IL-7, whereas only IL-7 was significantly different in the subgroups composed by the cutoff scores. In contrast, the median split subgroups showed significant differences in the levels of IL-4 and IL-5 between the high and low symptom subgroups (Table 3).

## 4. Discussion

There were several important findings derived from this secondary analysis that may be used to inform future studies. Although we chose a well-described symptom cluster of fatigue, depression, pain, and sleep disturbance, a majority of the sample had very low symptom scores for both pain and sleep disturbances. This initial screening of the range and distribution of each symptom score is important to determine whether the a priori symptom cluster is actually observed in the study sample. As was the case for this analysis, symptoms with extremely low scores may need to be excluded. Assigning a clinical significant cut-off score for each symptom a priori may be used to increase the homogeneity of the sample in terms of their symptom experience. The drawback to this approach is that there can still be a wide range of variation within the subgroups that could hamper the ability to detect clinically significant biological mechanisms.

Advantages of using the composite symptom cluster score include the ability to select highly interdependent symptom data, in this case between depression and fatigue, and incorporate multiple domains (intensity, frequency and interference) in the total score. The step of transforming each symptom scale to 0 - 100 composite score allowed equal representation of each domain as well as each symptom in the composite score. Measuring multiple domains of the symptom is consistent with a patient-centered approach to symptom measurement as a high intensity does not always translate to high interference. In contrast with the composite score technique, the cut-off score resulted in the inclusion of some symptom data that was less dependent while excluding others that were highly dependent. The median split technique resulted in the highest amount of variability in symptom scores and levels of significance in cytokines, suggesting this approach may increase the risk of Type I errors.

With an increasing focus of research on identifying the biological basis of symptom clusters in women with breast cancer, it would be advantageous to have one definition of a symptom cluster that is implemented across studies. Using a composite symptom cluster score or cut-off score to identify subgroups is an alternative until consensus on a precise definition of a symptom cluster can be reached. Both composite and cut-off score techniques provide a way to identify and compare women who have the most severe with the least severe symptoms, which can increase the likelihood of detecting clinically significant biological mechanisms.

## 5. Conclusion

Research studies focused on identifying clinically relevant biological mechanisms of symptom clusters experienced by individuals with cancer often use an a priori approach to symptom assessment. It is imperative to initially assess the range and distribution of each symptom score of the study sample to determine if the symptom cluster is indeed observed in the study sample. The best means for identifying subgroups is through using a composite score or assigning a clinically relevant cut-off score, so that the analysis may be performed among groups with the most similar symptom pattern.

## Figures and Tables

**Table 1:** Clusters defined by composite scores

Cluster							

	Low (N = 25)			High (N = 25)			t-test

	Mean	Std Dev	Std Err	Mean	Std Dev	Std Err	*p*-value
IL-1β	1.61	4.51	0.94	2.84	8.00	1.60	0.9230
IL-2	34.69	60.10	12.53	30.46	45.12	9.02	0.8865
IL-4	1.25	1.06	0.22	1.14	1.60	0.32	0.1322
IL-5	1.38	1.58	0.33	1.93	3.50	0.70	0.9325
IL-6	8.99	8.15	1.70	20.02	24.42	4.88	0.0324
IL-7	6.66	13.27	2.77	13.99	22.95	4.59	0.0158
IL-8	8.65	6.49	1.35	9.01	4.82	0.96	0.1721
IL-10	3.67	4.13	0.86	7.48	8.40	1.68	0.0761
IL-12	12.81	16.58	3.46	24.72	46.90	9.38	0.7911
IL-13	22.89	39.11	8.16	15.51	36.64	7.33	0.3424
IL-17	35.58	53.26	11.11	23.65	34.04	6.81	0.8480
G-CSF	22.82	30.40	6.34	14.09	12.30	2.46	0.7974
GM-CSF	222.74	253.73	52.91	258.77	212.64	42.53	0.5563
IFN-γ	96.70	149.83	31.24	127.25	301.99	60.40	0.6948
MCP-1	59.51	41.28	8.61	74.28	47.30	9.46	0.5060
MIP-1β	106.49	65.48	13.65	139.02	87.86	17.57	0.1109
TNF-α	15.01	28.02	5.84	23.10	57.18	11.44	0.5872

A composite symptom cluster score was computed for each individual by summing the two transformed scores. The top 20% of the sample with the highest scores compose the high cluster group whereas the lowest 20% of the sample with the lowest composite symptom score comprised the low symptom cluster group.

**Table 2:** Cluster defined by cutoff scores

Cluster							

	Low (N = 104)			High (N = 24)			*t-test*

	Mean	Std Dev	Std Err	Mean	Std Dev	Std Err	*p-value*
IL-1β	1.71	4.60	0.98	2.93	8.00	1.60	0.8479
IL-2	35.19	61.24	13.06	29.81	42.45	8.49	0.6497
IL-4	1.34	1.22	0.26	1.19	1.61	0.32	0.2133
IL-5	1.43	1.59	0.34	2.06	3.58	0.72	0.8178
IL-6	10.47	9.66	2.06	19.54	24.50	4.90	0.0895
IL-7	6.88	13.54	2.89	14.80	22.74	4.55	0.0087
IL-8	8.45	6.52	1.39	8.39	5.29	1.06	0.4890
IL-10	3.25	4.15	0.88	7.25	8.60	1.72	0.0791
IL-12	13.41	16.74	3.57	26.18	46.54	9.31	0.5969
IL-13	22.25	40.18	8.57	9.99	24.04	4.81	0.5094
IL-17	32.58	53.97	11.51	15.80	17.82	3.56	0.9352
G-CSF	26.56	34.04	7.26	15.29	12.85	2.57	0.8618
GM-CSF	165.24	166.03	35.40	268.07	207.49	41.50	0.1076
IFN-γ	111.88	164.26	35.02	123.00	303.79	60.76	0.7644
MCP-1	55.67	39.49	8.42	80.83	45.64	9.13	0.1893
MIP-1β	114.09	71.12	15.16	146.65	91.85	18.37	0.1634
TNF-α	18.47	30.40	6.48	22.18	57.46	11.49	0.8247

Cut-off scores for each symptom assessment measure were derived from the literature and were correlated with moderate to severe symptom intensity, frequency, and interference. The sample that met the cut-off scores of both fatigue and depression comprised the high cluster group whereas the sample that did not meet both symptom cut-off scores comprised the low cluster group.

**Table 3:** Cluster defined by median split

Cluster							

	Low (N = 63)			High (N = 65)			*t-test*

	Mean	Std Dev	Std Err	Mean	Std Dev	Std Err	*p-value*
IL-1β	2.18	5.72	0.74	1.79	5.21	0.66	0.0896
IL-2	45.02	98.28	12.69	33.27	57.66	7.26	0.3288
IL-4	1.45	1.38	0.18	1.13	1.42	0.18	0.0185
IL-5	2.32	3.13	0.40	1.80	3.09	0.39	0.0237
IL-6	15.25	18.73	2.42	16.46	19.19	2.42	0.5199
IL-7	5.59	9.48	1.22	17.97	65.89	8.30	0.1274
IL-8	10.00	10.39	1.34	10.62	11.46	1.44	0.4095
IL-10	4.67	4.45	0.57	5.42	6.36	0.80	0.6517
IL-12	27.19	82.93	10.71	19.40	32.70	4.12	0.5888
IL-13	39.00	75.27	9.72	18.68	31.65	3.99	0.0761
IL-17	44.44	70.53	9.11	33.03	66.63	8.39	0.2949
G-CSF	28.57	40.04	5.17	20.71	31.11	3.92	0.1419
GM-CSF	296.79	326.48	42.15	221.12	223.02	28.10	0.3069
IFN-γ	166.65	422.43	54.54	112.30	224.75	28.32	0.5844
MCP-1	61.06	38.82	5.01	64.60	40.51	5.10	0.6477
MIP-1β	119.33	66.65	8.60	129.50	74.12	9.34	0.5175
TNF-α	32.84	103.24	13.33	18.02	43.31	5.46	0.0694

A median split was applied to the sample based on the fatigue and depression scores with the top 50% with the highest symptom scores comprised the high cluster and the 50% with the lowest symptom scores comprised the low cluster group.
